# Southwestward Expansion of the Pacific Sleeper Shark’s (*Somniosus pacificus*) Known Distribution into the South China Sea

**DOI:** 10.3390/ani14152162

**Published:** 2024-07-25

**Authors:** Han Tian, Junsheng Zhong, Jiangyuan Chen, Yane Jiang, Jun Zhang, Wei Xie, Zuyuan Gao, Yuchao Wang, Haozhen Liu, Sujing Wang, Fei Zhang, Jie Yang, Kedong Yin

**Affiliations:** 1 School of Marine Sciences, Sun Yat-sen University, and Southern Marine Science and Engineering Guangdong Laboratory (Zhuhai), Zhuhai 519082, China; tianh53@mail2.sysu.edu.cn (H.T.); xiewei9@mail.sysu.edu.cn (W.X.); zgaoar@connect.ust.hk (Z.G.); wangyuchao@sml-zhuhai.cn (Y.W.); liuhaozhenzhen@foxmail.com (H.L.); 2 Shanghai Universities Key Laboratory of Marine Animal Taxonomy and Evolution, College of Fisheries and Life Science, Shanghai Ocean University, Shanghai 201306, China; jszhong@shou.edu.cn; 3 College of Fisheries, Ocean University of China, Qingdao 266003, China; megalodoneric@126.com; 4 South China Sea Fisheries Research Institute, Chinese Academy of Fishery Sciences, Key Laboratory for Sustainable Utilization of Open-Sea Fishery, Ministry of Agriculture and Rural Affairs, Guangzhou 510300, China; jianglinhui3@aliyun.com (Y.J.); zhangjun@scsfri.ac.cn (J.Z.); 5 Institute of Acoustics of the Chinese Academy of Sciences, Beijing 100190, China; wangsujing@mail.ioa.ac.cn (S.W.); zhangfei@mail.ioa.ac.cn (F.Z.); yangjie@mail.ioa.ac.cn (J.Y.)

**Keywords:** *Somniosus pacificus*, subgenus *Somniosus*, habitat expansion, deep-sea baited observations, South China Sea

## Abstract

**Simple Summary:**

Our study provides the first record of Pacific sleeper sharks in 1629 m deep waters off the southeast coast of Hainan Island in the South China Sea. At a site, we placed a dead cow as bait and a metal-framed cage with cameras and observed eight individuals and their feeding activities by using ROV diving. The record indicates an expansion of the known distribution range of the Pacific sleeper sharks to the west of the northern South China Sea. Such a finding demonstrates that the habitat ranges of large animals are far wider in deep seas than we know from observations in the upper layer, and habitat expansion of some animal populations may have already taken place under intensive anthropogenic influences such as climate changes.

**Abstract:**

We conducted an experiment of planting a dead cow and a metal-framed cage with cameras on the 1629 m deep sea floor off the southeast coast of Hainan Island in the northwestern South China Sea, using ROV diving and setting up a video camera on the cage to observe animals who came to eat the bait. The deep-sea cameras captured footage of eight Pacific sleeper sharks (*Somniosus pacificus*) swimming and feeding around the dead cow. To our knowledge, this is the first time the occurrence of such a shark species has been reported in the South China Sea. Eight individuals were differentiated based on the characteristic differences displayed in the images, with lengths of 1.9 to 5.1 m estimated. The video camera also recorded the predators’ behavior of tearing at the dead cow on the seabed. It was discovered that Pacific sleeper sharks are not strictly solitary and exhibit queue-feeding behavior. This study is significant as it documents a record of a data-scarce shark species, for which little information is available in the literature. It also documents an expansion of the species’ known habitat from the north Pacific Ocean into the South China Sea. Such sharks diving into the deep sea to predate on dead animals also suggests that occurrences of large chunks of dead organic bodies falling onto the deep sea might have been more frequent than we previously thought in the South China Sea. The findings have implications for understanding the geographic connectivity of large swimming animals between the South China Sea and the Pacific Ocean and provide scientific evidence for formulating conservation and management strategies for sharks and other large animals in the oceans.

## 1. Introduction

The vast dark and deep water in the world’s oceans was almost unexplored and its life was unknown before the development of deep-sea vehicles. Deep-sea dives by underwater vehicles such as ROVs and HOVs have opened the door to the discovery of many unknown animals in the deep ocean. Deep-sea meat-bait experiments were found to be an effective means to attract deep-sea organisms and to promote prolonged and continuous visual observations, which fills the void of sampling deep-sea mobile animals left by traditional fisheries. The sample technique mitigates the substantial randomness associated with single diving observations and circumvents the typical avoidance response exhibited by fish, thereby facilitating visual observations of species aggregation. Since the HOV Alvin discovered the first whalefall in the deep sea off California in 1987, quite a few whalefall or cowfall experiments have been conducted [1,2]. Recently, following the first discovery of a whalefall in a seamount in the South China Sea (SCS) [3], cowfall experiments were conducted, in which three dead cows were planted at three different depths on the slope of a mountain in the SCS [4]. Such an approach has significantly advanced our understanding of the biodiversity distribution of rare, medium- to large-sized organisms [5,6]. These imaging data serve as the most direct evidence for the ecological dispersal of deep-sea species and provide crucial information for their geographic expansion [7].

Sleeper sharks are one of the most enigmatic shark species in oceans and are renowned for their exceptional longevity and prolonged periods of deep-sea habitation, belonging to the class Chondrichthyes, order Squaliformes, family Somniosidae, and genus *Somniosus* [8,9]. The genus *Somniosus* currently comprises six nominal species in two subgenera, *Somniosus* and *Rhinoscymnus*, with three nominal species in each subgenus [10]. The two subgenera exhibit significant differences in body size. Species within the subgenus *Somniosus* are generally larger, with the largest length exceeding four meters. These species include the Greenland shark *S. (S.) microcephalus* in the Arctic and North Atlantic, the Antarctic sleeper shark *S. (S.) antarcticus* in the Southern Hemisphere, and the Pacific sleeper shark *S. (S.) pacificus* in the Arctic and North Pacific [10]. In contrast, species within the subgenus *Rhinoscymnus* are smaller, with the largest length not exceeding 1.5 m. These species include the frog shark *S. (R.) longus* in the western Pacific, the little sleeper shark *S. (R.) rostratus* in the eastern North Atlantic and Mediterranean Sea (which has been reported to be a synonym of the previously named *S. (R.) bauchotae*) [11], and *S. (R.) cheni* in the Pacific Ocean off Taiwan [12]. Regarding the geographical distribution of these known species, *Yano* et al. found that each species is largely confined to specific geographic regions [10]. Although morphological distinctions among the three species within the subgenus *Somniosus* have been delineated by *Yano* et al. [10], the classification of the three species within the subgenus *Somniosus* may be subject to a revision in the future with the advancement of DNA technology in recent years [13,14,15,16].

Till now, the Pacific sleeper shark *S. pacificus* Bigelow & Schroeder 1944 remains a species whose distribution has not been fully deciphered [17]. Traditionally, the primary distribution area of *S*. *pacificus* was considered to be confined to the North Pacific with a certain established population size [18,19,20]. Studies over the past decade gradually revealed the possibility of a wider distribution range for *S. pacificus* and its certain ecological habits, including high-latitude areas. The shark is known for its diel vertical migration, varying swimming speeds at different depths, and confinement to deep-sea regions at lower latitudes (speculated to be at a depth limit of approximately 2000 m) [9,21,22,23]. In these reports, the physical specimens of *S. pacificus* primarily occur through incidental bycatches or strandings, while a few deep-sea observations have provided valuable video footage of *S. pacificus* [9,16,20]. However, close observations in the deep sea remain scarce, especially regarding adult individuals. There is a need for more research and monitoring to understand its population dynamics and ecological requirements, as the International Union for Conservation of Nature (IUCN) reclassified *S. pacificus* from “Data Deficient” to “Near Threatened” in 2021. This change does not imply that we have sufficient information about the species’ population. On the contrary, within known habitats, the population of this species has significantly declined (by 20–29%), suggesting that it faces severe survival pressures [24].

The SCS is the largest marginal sea in the western North Pacific and is considered a critical conduit linking the western Pacific with the Indian Ocean [25]. Currently, approximately 100 species of sharks are found in the SCS [26,27]. A literature review showed that the eastern waters of Taiwan (121.5° E, ~24° N) are the most southern area in the western Pacific where *S. pacificus* has been caught [28,29]. In 2023, *Claassens* et al. discovered the presence of this species during their cruise around the Solomon Islands and Palau using video imaging. The species was primarily observed at a depth of around 1000 m, indicating a significant southward expansion of its known distribution into lower latitudes [30]. Until now, there has been no documented report of the presence of other large sleeper sharks in the central area of the SCS. The objectives of this study are to report the first record of *S. pacificus* in the SCS and give a first-hand close observation of its behavior.

## 2. Materials and Methods

**Figure 1:**
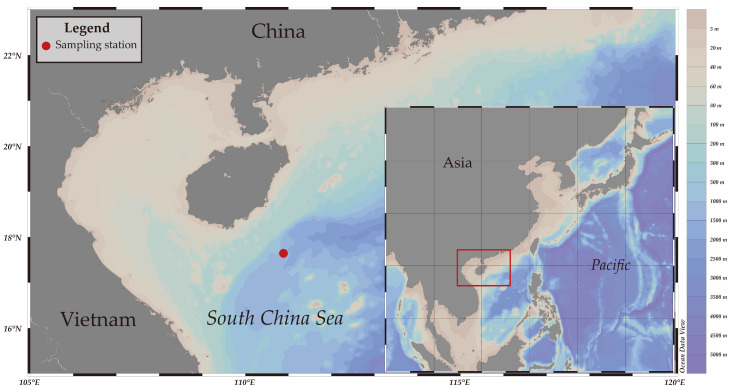
The sampling site on the seafloor of the South China Sea.

On 3 November 2022, a dead cow (*Bos taurus*) in two halves with internal organs removed and a metal-framed cage with cameras were placed on the seafloor at a depth of 1629 m (110°53′465″ E, 17°38′751″ N) in the waters southeast of Hainan Island in the SCS (Figure 1). The cow was processed at a certified slaughterhouse, subsequently further processed within the facility, and purchased as a meat product. In this study, the term “cow” is used in place of “meat” to facilitate readers’ understanding of the shark’s body size. The cage was placed on the seafloor from a vessel, and a remotely operated vehicle (ROV, FCV3000C, maximum operational depth of 3000 m) equipped with two mechanical arms (Titan 4 and Rigmaster, Schilling, Houston, TX, USA) was used to examine its proper position. The deep-sea observation system attached to the cage consisted of a camera and a video camera featuring a resolution of 4608 × 3456, a zoom range of 3.8–11 mm, an underwater light, and a timing control chamber (software: Ocean Data View v5.1.2; Photoshop2023). The electricity for all electronic devices was provided by an underwater electric cable laid on the seafloor operated by the Institute of Acoustics, Chinese Academy of Sciences. The cow and cage were arranged approximately two meters apart as the bait to attract hunting scavenging animals. The observations of the cage lasted from 3 November to 21 November 2022.

The cameras captured the morphological traits and details of multiple individuals of the genus *Somniosus*, which allowed their identification and distinctions from one another. We extracted details of shark morphological traits that validate their unique identities through simple video editing of images. We selected representative pictures to facilitate species identification and underscore individual variations. The images for verification included lateral views; head (including lateral, dorsal, and ventral views of the head); details of the mouth, pelvic fins, gills, and caudal fin; comparison shots with a reference object; and images of the side, pigment spots, parasites, or other characteristic features.

In this study, the morphological measurements included four easily identifiable parameters as described by Yano et al. [10]. These parameters include total length, the distance from the snout to the first dorsal fin (posterior origin, PO), the distance from the snout to the second dorsal fin origin, and the distance from the snout to the upper caudal fin origin. The 2.7 m long cow was used as a reference object to roughly estimate the shark length parameters from 2D video screenshots. Images were selected where the sharks appeared as close as possible to the left side of the cow, with their snouts either touching or actively biting the cow. Despite the consumption or displacement of the bait due to marine organism activity, the known total length of the cow was consistently used to estimate the lengths of different body parts of the sharks. To estimate the length of life of moving sharks, the observation method described by Smith et al. [31] was referenced and simplified according to the specific conditions of our experiment. The cow’s total length was divided into 100 segments of 2.7 cm each to measure the observed sharks, and the sharks’ lengths were estimated by counting these segments. Therefore, the estimated lengths were about 1.35 cm (half of each segment) different from the actual lengths, or the estimation might introduce errors of a few centimeters in the measurement. To minimize errors caused by subjective judgment, the measurements were recorded in meters and rounded to one decimal place. Length estimates were made using Image J 1.54g Java1.8.0_345 (https://imagej.net/software/imagej/) URL (accessed on 24 March 2024). These identifiable length features served as approximate estimation indicators and were compared with other species within the genus Somniosus. This comparison enhanced the scientific validity of the species identification. The high-resolution images and videos along with existing background knowledge of *Somniosus* species make it possible to identify the species without physical specimens.

## 3. Results and Discussion

### 3.1. Number and Differences of Individuals

During the 18-day period, we recorded a total of eight shark individuals assigned as No. 1 to No. 8 based on some distinct identifying features, as indicated in Table 1 with their appearances. From November 4 to 5, the first four individuals were observed successively. During 4:00–17:00 on 5 November, the animal bait was dragged out of the camera view by No. 4, and visuals of more sharks were lost. During this period, only one shark was observed swiftly appearing and disappearing within the filming area. Additionally, we saw sediment plumes in two instances, likely resulting from disturbances by shark-feeding behavior. Later on November 5th, the bait was reinstalled using the ROV, and a feeding shark was recorded during the reinstallation. Subsequently, two more sharks were documented in our records until 21 November.

**Figure 2:**
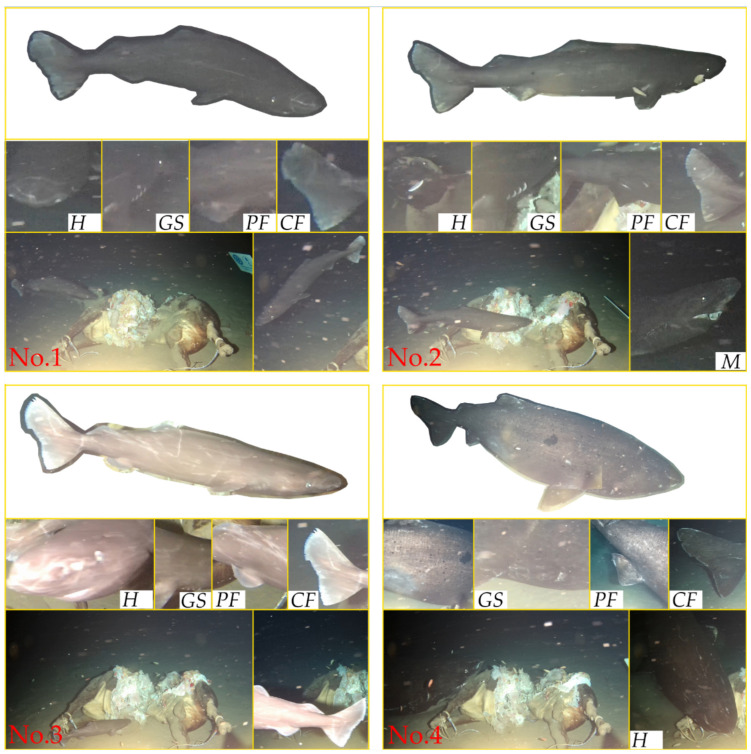
Observations of No. 1, 2, 3, and 4 Pacific sleeper sharks (*Somniosus pacificus*) in the South China Sea at 1629 m depth. *H*, head; *M*, mouth; *GS*, gill slit; *PF*, pelvic fin; *CF*, caudal fin.

**Figure 3:**
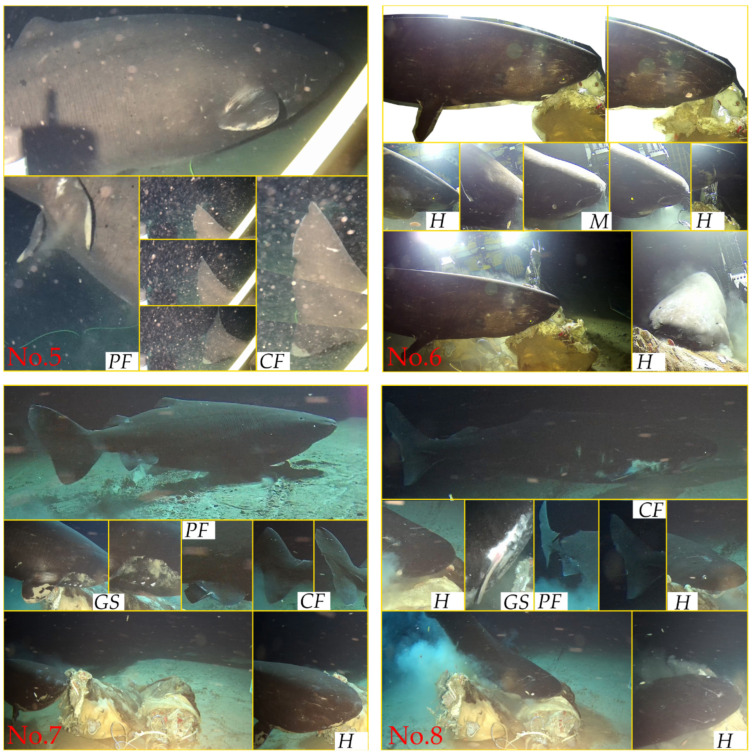
Observations of No. 5, 6, 7, and 8 Pacific sleeper sharks (*Somniosus pacificus*) in the South China Sea at 1629 m depth. *H,* head; *M,* mouth; *GS*, gill slit; *PF*, pelvic fin; *CF*, caudal fin.

**Figure 4:**
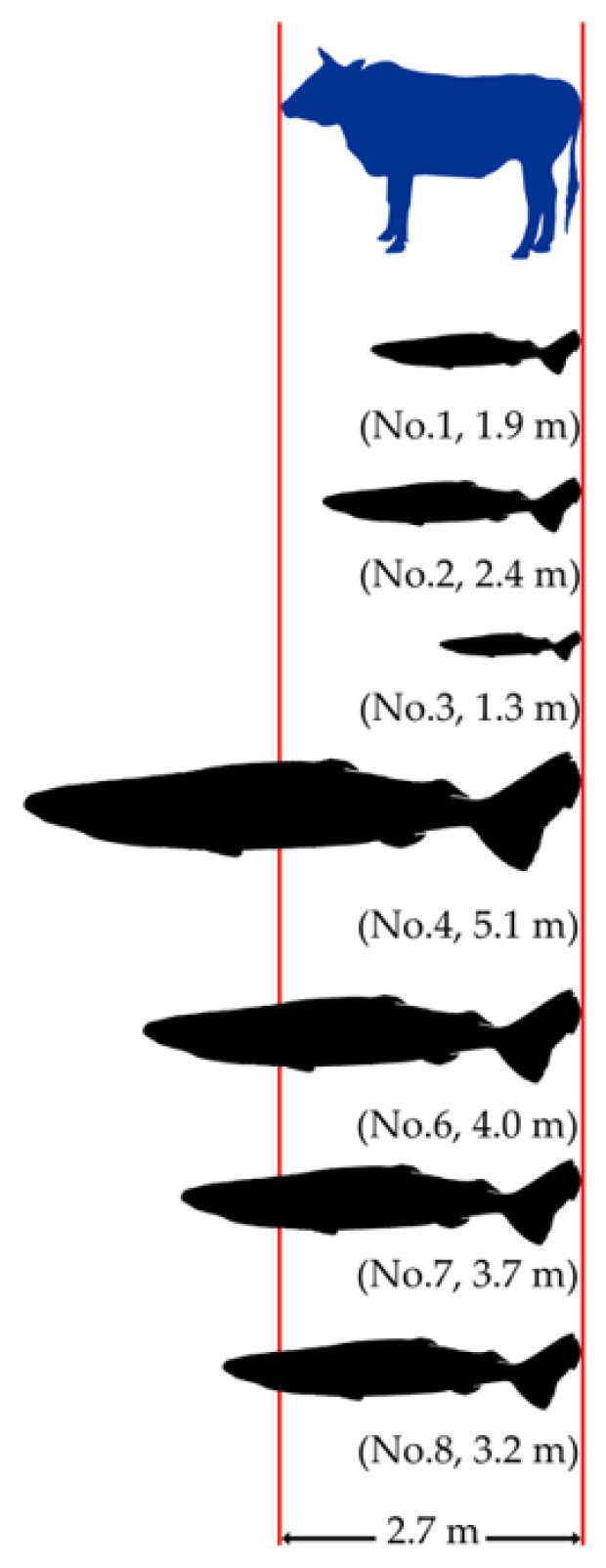
Estimation of specimens’ (*Somniosus pacificus*) total length based on the reference length of the cow.

In chronological order, eight individuals are further described in detail (Figure 2 and Figure 3). No. 1 is characterized by a long horizontal scratch along its flank, from the gill area to the base of the first dorsal fin, which is considered to be a temporarily immutable mark on the shark’s skin, with several scratches on its head. The estimated length of No. 1 is about 1.9 m. No. 2 has a black circular spot near the base of its pelvic fins and minor notches on its caudal fin. No. 2′s length is approximately equivalent to the cow’s torso and is estimated to be about 2.4 m. No. 3 is the smallest shark among the eight sharks, with an estimated length of 1.3 m. It is covered in numerous scratches and has two significant notches in its caudal fin. No. 4 is a significantly large individual and is estimated to be 5.1 m long. It is distinguished by extensive black patches, scattered small black spots along its flank, and a noticeable notch in its tail. No. 5 appears to be a large individual within the field of view and is differentiated from No. 4 by smaller and fewer spots. The visual of No. 6 during the ROV’s operation did not include its complete image, but its distinctive clustered scratches around the eye area differentiate it from all others except for No. 5. Considering the approximately seven-day interval between the records of No. 5 and No. 6, we distinguished and documented these two individuals separately, with No. 6′s length estimated at 4.0 m. Moreover, the behavior of individual No. 6 attacking the bait was captured, including the retraction of the eyes while biting the bait, and the auxiliary respiratory function of the spiracles, stirring up the resuspension of sediment. No. 7′s head and tail appear to have some parasites, distinguishing it from others, with a length of about 3.7 m. No. 8 (approximately 3.2 m) appears to have an injured pectoral fin, a significant distinguishing feature. Except for shark No. 5, which lacked the cow as a reference for body length measurement, the lengths of the other seven individuals were estimated and are summarized in Figure 4. It should be noted that the unclear video screenshots and the sharks’ movement or image distortion caused inaccurate length estimates.

### 3.2. Species Identification

The whole bodies of No. 5 and No. 6 did not appear within the field of view, resulting in the loss of some characteristic descriptions. All individuals exhibit a high degree of morphological consistency identifiable through a detailed examination of the video footage, and several common characteristics. They possessed robust cylindrical bodies, with short and blunt snouts featuring prominently large nostrils on the top. Each individual has five pairs of gill slits, relatively small eyes, and a pair of distinctive spiracles located behind the eyes. They also have two prominent dorsal fins located in the middle to the rear part of the body and lack spines in front of the dorsal fins. The caudal fin is heterocercal, with the upper lobe slightly longer than the lower lobe, accompanied by a short caudal peduncle. Based on these visually discernible descriptive references, these individuals are identified as the genus *Somniosus* [10,11,32].

Further identification of sharks from the genus *Somniosus* needs to be based on body length, body proportion references, distribution area, and other detailed physical characteristics [10]. The estimated lengths of the subdivided body parts for all observed individuals are in Table 2, and the percentage ratios to the estimated total lengths are in Table 3. A total length greater than 1.5 m is considered an important distinguishing marker between the two subgenera within the genus *Somniosus*, which has been widely accepted in studies of this genus [10,16]. In our observations, only No. 5 could not be assessed for body length due to the lack of a reference object (the cow) in the field of view. However, based on the available photographic evidence, No. 5 can be preliminarily judged as a robust and large individual (with a body length far greater than 1.5 m). This means that among the eight individuals, only No. 3 has a total length of less than 1.5 m (estimated at 1.3 m). Therefore, in the current state of limited research progress on the maximum body length of the subgenus *Rhinoscymnus* species, seven large individuals, out of eight, are classified as belonging to the subgenus *Somniosus*.

We compared the percentage of the total length of shark No. 3 with known species from the subgenus *Rhinoscymnus* (Table 4). Our comparison suggests that, based on our estimated lengths, the distance between the snout and first dorsal origin (PO) of No. 3 significantly differs from that of other individuals in the subgenus *Rhinoscymnus* (*p* < 0.05). Comparing the morphology of other known subgenus *Rhinoscymnus* specimens with No. 3 shows three significant differences which are visibly noticeable: (1) the first dorsal origin (PO) is more anterior, (2) the first dorsal fin is larger than the second dorsal fin, and (3) the distance between the first dorsal origin (PO) and second dorsal origin is greater. In addition, the images and length relationships of the observed individuals, serving as evidence, show no significant differences between individuals (*p* > 0.05). Based on this evidence, the observed individuals of the genus *Somniosus* cannot be matched with the biological characteristics of the subgenus *Rhinoscymnus*, and therefore, they are considered to be species of the subgenus *Somniosus*.

To further determine the identification of the species, the body length ratios of the observed sharks are compared with those of three species within the subgenus *Somniosus* (*S.* (*S.*) *microcephalus*, *S.* (*S.*) *antarcticus*, and *S.* (*S.*) *pacificus*), as presented in Table 5. However, accurate conclusions could not be drawn based on these parameters. Yano et al.‘s study similarly suggested a degree of morphological overlap among the three *Somniosus* species, making identification based on a few morphological traits challenging [10]. Typically, species identification within the subgenus *Somniosus* requires an examination of external appearance, habitat, and internal structures or features (e.g., rows of teeth in the upper and lower jaws, spiral valve ring count, and vertebral count). In terms of external morphology, *Yano* et al. identified the distance between dorsal fins as a distinguishing feature for *S.* (*S.*) *microcephalus* compared to the other two species, noting that *S.* (*S.*) *microcephalus* exhibits a dorsal fin spacing nearly equal to the length of the pre-gill slit (102.5% of the pre-gill slit length), whereas in the other two species, the dorsal fin spacing is less than the pre-gill slit length (73.2% in *S.* (*S.*) *pacificus* and 82.2% in *S.* (*S.*) *antarcticus*, with slight overlap between the latter two) [10]. Observations extracted from video footage (No. 1, No. 2, No. 3, No. 4, No. 7, and No. 8) show that the dorsal fin spacing and pre-gill slit length were both less than the pre-gill slit length, despite the difficulty in quantifying these observations and differentiating between the 73.2% and 82.2% ratios. To prevent further confusion regarding species relationships, *Yano* et al. concluded that the three *Somniosus* species were each restricted to specific regions: the North Atlantic (*S.* (*S.*) *microcephalus*), the North Pacific (*S.* (*S.*) *pacificus*), and the Southern Hemisphere (*S.* (*S.*) *pacificus*) and adjacent seas [10]. This implies that the eight *Somniosus* individuals observed are highly unlikely to be *S.* (*S.*) *microcephalus* and are most likely *S.* (*S.*) *pacificus* according to the current classification methods. This challenge is also highlighted in the review by *Matta* et al. [16]. Multiple investigators have concluded that there is not sufficient genetic variation in mitochondrial or nuclear DNA to distinguish between *S.* (*S.*) *pacificus* and *S.* (*S.*) *antarcticus*, suggesting that they comprise a single species ranging throughout the Pacific Ocean, warranting revision of the taxon [13,14,15], despite the morphometric differences noted by Yano et al. [10]. Therefore, given the ongoing controversy regarding the classification of *S.* (*S.*) *pacificus* and *S.* (*S.*) *antarcticus*, and considering the geographical proximity of the SCS to the North Pacific, the sharks observed in this study are *S. pacificus* based on the aforementioned perspectives. In this study, it is difficult to determine the sex of the sharks as there is a lack of anatomical information in our research specimens. However, based on our observation, we speculate that all the individuals seem to be female, based on the identification method which involves distinguishing the shape of the pelvic fins (claspers are not prominent), described by Yano et al. and Matta et al. [10,16].

**Figure 5:**
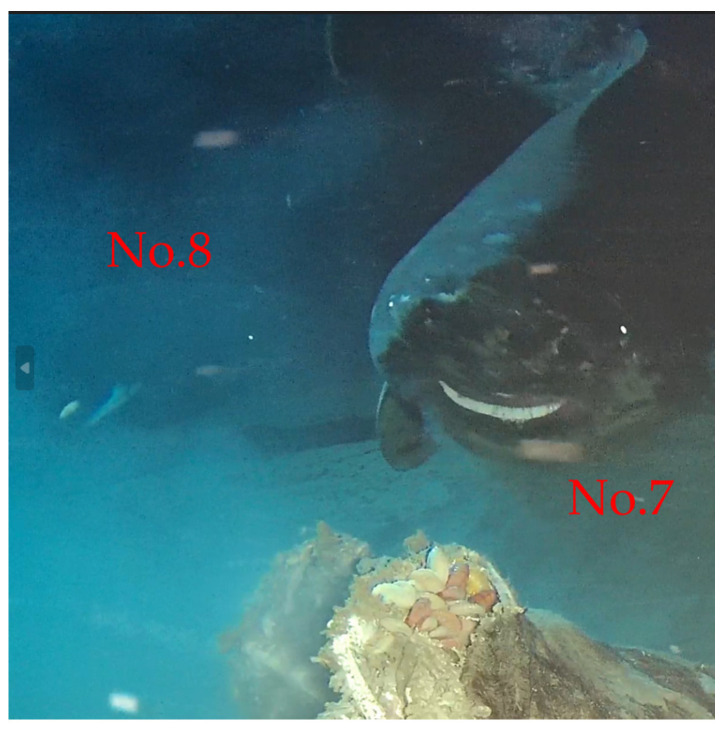
Records of two *Somniosus pacificus* individuals (No. 7 and 8) appearing simultaneously and feeding in a queue in the South China Sea at 1629 m depth.

No. 7 and No. 8 appeared successively (Figure 5). The successive appearances of two sharks present an intriguing phenomenon. Unfortunately, we cannot ascertain their relationship. Records of multiple *S. pacificus* appearing simultaneously are extremely rare [30,34,35]. Thus, their queue-like or sequential feeding behavior emerges as a fascinating activity that helps prevent direct conflict among large predators. This conclusion is based on the assumption that these sharks typically operate as solitary individuals [36]. An alternative explanation might also be plausible: within a group, these sharks may exhibit a social hierarchy. This scenario presents numerous narratives and scientific questions ripe for exploration in the future. Therefore, our research lays the foundation for monitoring the population dynamics of the Pacific sharks in the SCS.

### 3.3. An Expansion of the Known Distribution Range of Somniosus Pacificus

Our study compiles reports of new distribution areas for *S. pacificus* and suspected *S. pacificus* from the IUCN Red List of Threatened Species 2021 [24] (data collected up to August 2019) to the present (Table 6). Supported by our evidence, the distribution range of *S. pacificus* has expanded westward by over 1200 km and southward by over 730 km (121.5°E, ~24°N), officially moving from the fringes of the Northwest Pacific into the core region of the SCS [10,12,28,29,32]. If the Pacific sleeper sharks we observed migrated from their typical breeding grounds in the Northwest Pacific, considering the relatively shallow nature of the Taiwan Strait, their most probable route into the SCS would be through the Bashi Channel. Based on the observation of eight sharks of varying sizes at the same location, we speculate that the population of Pacific sleeper sharks in the SCS is larger than what we have observed. However, to date, there have been no related sighting reports. The lack of this shark’s records does not imply a shift in habitat use and it is perhaps simply due to the absence of sightings because the deep-sea fish trawling surveys conducted in the SCS do not usually reach the water below 1000 m where Pacific sleeper sharks are active [37,38].

Investigating the distribution and ecological habits of deep-sea organisms in the SCS remains a significant challenge due to the limited number of observations. This limitation impedes our ability to determine whether these deep-sea organisms can establish their habitats in this region or make excursions to this region. Our observation of this shark suggests an expansion of the known distribution range of the species. The similar whalefall experiments conducted at about 1600 m and 2300 m on the slope of a seamount in the middle of the SCS (113~116° E, 12~14° N) [4] showed some other sharks, not this genus *Somniosus*. Recognizing the gap in knowledge regarding deep-sea shark species, such as the Pacific sleeper shark, it is crucial to develop targeted conservation plans [40,41]. These efforts should aim to investigate and sustain the population of Pacific sleeper sharks in the SCS.

## 4. Conclusions

Our study documented the presence of eight Pacific sleeper sharks (*S. pacificus*) at a depth of 1629 m in the SCS using the bait of a dead cow and making continuous observations powered by deep-sea floor cables. The presence of *S. pacificus* in the central area of the SCS suggests the expansion of the known distribution range of *S. pacificus* from the north Pacific Ocean. It also highlights their adaptability to the deep-water environment and their habitation along the continental plain in the SCS. Observations of their feeding behavior and social interactions have provided deeper insights into their ecological roles and behavioral patterns. This study demonstrates that many aspects of the biodiversity and ecosystem dynamics of deep-sea environments remain to be discovered. These findings are crucial for developing informed conservation strategies and management practices for the Pacific sleeper shark to effectively protect this mysterious deep-sea inhabitant and other species.

## Figures and Tables

**Table 1:** Number and behavior description of *Somniosus pacificus* individuals which were sighted in November 2022.

Sighting Individuals	Sighting Time (H: M)	Appearance or Disappearance	Procedure Description	Stage
No. 1	4 November 12:21	1st appearance	Patrolling and eating	Stage 1
	4 November 12:36	1st disappearance		
	4 November 12:43	2nd appearance	Patrolling and eating	
	4 November 12:54	2nd disappearance		
No. 2	4 November 19:40	1st appearance	Patrolling and eating	
	4 November 19:51	1st disappearance		
	4 November 19:57	2nd appearance	Patrolling and eating	
	4 November 20:11	2nd disappearance		
No. 3	4 November 23:39	1st appearance	Patrolling but leaving	
		1st disappearance		
	4 November 23:45	2nd appearance	Patrolling but leaving	
		2nd disappearance		
	4 November 23:48	3rd appearance	Patrolling and eating	
	4 November 23:52	3rd disappearance		
No. 4	5 November 03:07	1st appearance	Patrolling and eating	
	5 November 04:47	1st disappearance		
	5 November 04:53		Smoke raging	
No. 5	5 November 08:38		Smoke raging	Bait missing
	5 November 08:57	Appearance and disappearance	Flashing	
No. 6	5 November 15:42	-	Eating	ROV debugging (bait reposition)
	5 November 15:57	-		
No. 7	5 November 17:09	Appearance	Patrolling and eating	Stage 2
	5 November 17:41	Disappearance		
No. 8	5 November 17:41	Appearance	Patrolling and eating	
	5 November 17:57	Disappearance		

‘-’ means loss of information.

**Table 2:** Length estimates of *Somniosus pacificus* observed in the South China Sea.

Measurements	No. 1	No. 2	No. 3	No. 4	No. 5	No. 6	No. 7	No. 8
Total length (m)	1.9	2.4	1.3	5.1	-	4.0	3.7	3.2
Snout tip to:								
14 1st dorsal origin (PO)	0.9	1.1	0.6	2.5	-	-	1.7	1.4
15 2nd dorsal origin	1.4	1.7	0.9	3.7	-	-	2.4	2.1
16 upper caudal origin	1.6	2.1	1.1	4.4	-	-	3.0	2.7

‘-’ means loss of information.

**Table 3:** Proportional dimensions expressed as the estimated percentage of the total length for *Somniosus pacificus* observed in the South China Sea.

Measurements	No. 1	No. 2	No. 3	No. 4	No. 5	No. 6	No. 7	No. 8
Total length (m)	1.9	2.4	1.3	5.1	-	4.0	3.7	3.2
In % of total length Snout tip to								
14 1st dorsal origin (PO)	47.4	45.8	46.2	49.0	-	-	45.9	43.8
15 2nd dorsal origin	73.7	70.8	69.3	72.5	-	-	64.8	65.7
16 upper caudal origin	85.2	87.5	84.7	86.2	-	-	81.0	84.5

‘-’ means loss of information.

**Table 4:** Comparison of the proportional dimensions expressed as the estimated percentage of the total length for shark No. 3 and species from subgenus *Rhinoscymnus* (*Somniosus* (*Rhinoscymnus*) *longus*; *Somniosus* (*Rhinoscymnus*) *rostratus*; *Somniosus* (*Rhinoscymnus*) *cheni*).

Measurements	No. 3 (*n*)	*S.* (*R.*) *longus* (*n*)	*S.* (*R.*) *rostratus* (*n*)	*S.* (*R.*) *cheni* (*n*)
Data Sources		[10]	[33]	[12]
Total length (m)	1.3 (1)	1.30 (1)	1.43 (1)	1.34 (1)
In % of total length Snout tip to				
14 1st dorsal origin (PO)	46.2 (1)	36.6 (1)	35.9 (1)	36.4 (1)
15 2nd dorsal origin	69.3 (1)	67.4 (1)	66.2 (1)	68.6 (1)
16 upper caudal origin	84.7 (1)	81.6 (1)	80.7 (1)	82.5 (1)

‘(*n*)’ means the number of specimens.

**Table 5:** Comparison of the proportional dimensions expressed as the estimated percentage of the total length for observed sharks in the present study and species from subgenus *Somniosus* (*Somniosus* (*Somniosus*) *pacificus*; *Somniosus* (*Somniosus*) *antarcticus*; *Somniosus* (*Somniosus*) *microcephalus*).

Measurements	Present Study (*n*)	*S.* (*S.*) *pacificus* (*n*)	*S.* (*S.*) *antarcticus (n)*	*S.* (*S.*) *microcephalus* (*n*)
Data Sources		[10]	[10]	[10]
Total length (m)	1.90~5.10 (7)	0.42~4.30 (29)	0.94~4.38 (23)	0.65~4.80 (38)
In % of total length Snout tip to				
14 1st dorsal origin (PO)	43.8~49.0 (6)	44.2~52.6 (25)	45.0~53.1 (18)	35.5~45.9 (37)
15 2nd dorsal origin	64.8~73.7 (6)	65.8~70.7 (29)	54.0~78.1 (22)	62.0~71.0 (37)
16 upper caudal origin	81.0~87.5 (6)	78.0~84.1 (29)	78.0~92.4 (23)	75.4~86.8 (38)

‘(*n*)’ means the number of specimens.

**Table 6:** New reports on suspected *Somniosus pacificus* since 2019.

Species Names Used in the Article	Recording Method	Time (Y/M/D)	Area	Location		Depth (m)	References
*Somniosus* cf. *pacificus*	Video	January 2015	Solomon Islands	~158°15′ E	~8°50′ S	937	[30]
*Somniosus* cf. *pacificus*	Video	January 2021	Palau	~134°32′ E	~7°13′ N	1288	[30]
*Somniosus pacificus*	Video	21 July 2016	Suruga Bay	138°23′ E	34°52′ N	608.6	[23]
*Somniosus pacificus*	Video	21 July 2016	Suruga Bay	138°23′ E	34°51′ N	602.9	[23]
*Somniosus pacificus*	Video	29 September 2016	Suruga Bay	138°32′ E	34°48′ N	962.4	[23]
*Somniosus pacificus*	Video	19 April 2017	Suruga Bay	138°25′ E	34°52′ N	542.2	[23]
*Somniosus* sp.	Capture	22 April 2022	Glover’s Reef Marine Reserve	~−87°47′ E	~16°44′ N	~350	[39]

## Data Availability

The data and additional images in the article can be provided upon obtaining consent from the corresponding author, by reaching out to the first author.
